# PCOS and Role of Cumulus Gene Expression in Assessing Oocytes Quality

**DOI:** 10.3389/fendo.2022.843867

**Published:** 2022-05-26

**Authors:** Nurainie Sayutti, Muhammad Azrai Abu, Mohd Faizal Ahmad

**Affiliations:** ^1^Department of Obstetrics and Gynaecology, National University of Malaysia, Kuala Lumpur, Malaysia; ^2^Reproductive Centre, Hospital Chancellor Tuanku Muhriz, National University of Malaysia, Kuala Lumpur, Malaysia

**Keywords:** polycystic ovarian syndrome, review, infertility, assisted reproductive technique, oocyte quality, cumulus cells gene expression

## Abstract

The global infertility rate has been declining from year to year. PCOS is one of the treatable accountable causes contributing to anovulatory infertility. Nevertheless, the success rate of treatments and live-birth outcomes especially involving assisted reproductive techniques is still not very promising. There is a reduction in the development potential of oocytes and high-quality embryos in PCOS patients compared to non-PCOS patients. A critical step in IVF treatment is the assessment of oocyte and embryo competence before embryo transfer. Oocytes in metaphase II are very fragile. Repeated morphological assessment on these oocytes may directly impair the quality and affect the whole process. Identification of potential biomarkers especially in the cumulus cells oocytes complex will help to predict the outcome and may create space for improvement. This review has explored gene expression in cumulus cells with regards to oocytes quality in both normal and PCOS women. The gene expression was classified according to their physiological function such as the contribution on cumulus expansion, cumulus cells apoptosis, and glucose metabolism. Collectively, the review suggested that positive expression of HAS2, PTX3, GREM1, and VCAN may correlate with good quality oocytes and can be used as an indicator among PCOS women.

## Introduction

Polycystic Ovarian Syndrome is a complex multi-spectrum disease involving the endocrine, metabolic and reproductive systems with an incidence of 6-21% (1). The Rotterdam clearly described the diagnostic criteria of the disease: 1) rare ovulation or lack of ovulation, 2) excessive activity of androgens confirmed by a clinical or laboratory examination, and 3) features of polycystic ovaries in the ultrasound after exclusion of other pathologies. The presentation can be from asymptomatic to having a full-blown disease. It includes menstrual disturbance, infertility, and metabolic disorders such as insulin resistance, diabetes, and obesity. Anovulatory infertility is common among PCOS women and serves as one of the reasons for assisted reproductive techniques (ART) in this specific group. Despite having a good number of follicles, the number of good competence oocytes is scarce. A good quality oocyte means it has a high level of intrinsic ability to undergo meiotic maturation, fertilization, proper embryonic development, successful pregnancy (2). This review is intended to describe the importance of cumulus cells gene expression in oocyte maturation, and their impact on fertility among PCOS women.

## Basis of Folliculogenesis and Its Disturbances in PCOS

**Figure 1:**
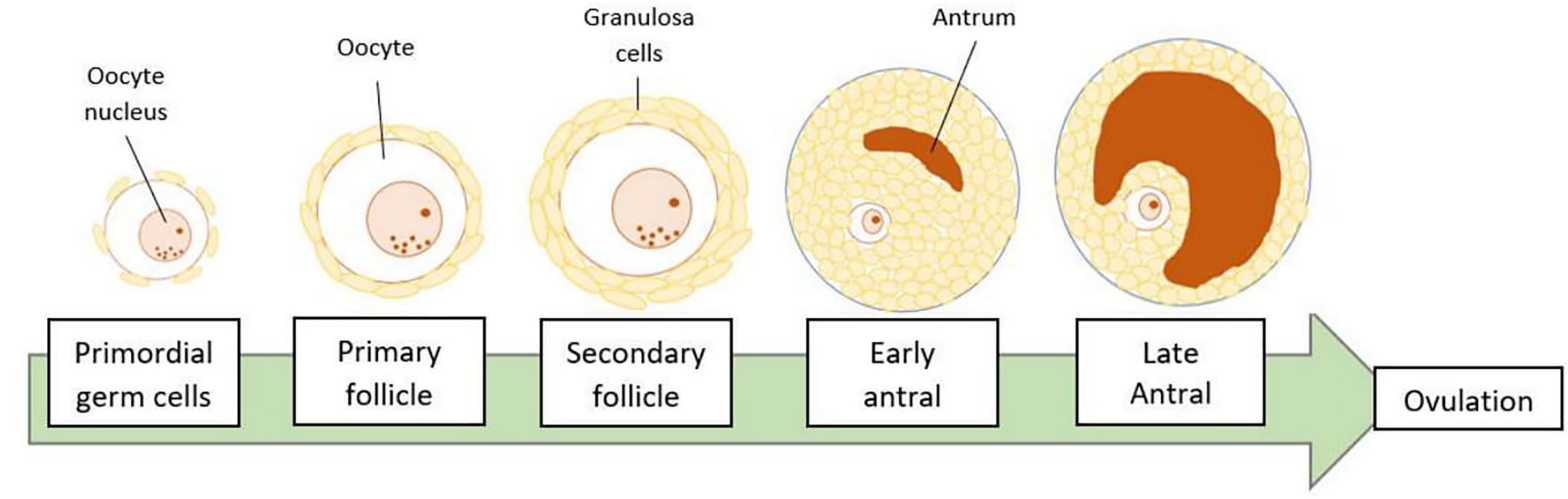
Stages of folliculogenesis. The ovarian follicle will go through primordial, primary, secondary, antral, and pre-ovulatory or late antral developmental stages. Finally, an oocyte is released during ovulation, leaving the remaining ruptured follicle, known as the corpus luteum.

Folliculogenesis refers to the pathway of ovarian follicle development from primordial germ cells to become mature oocytes (**Figure 1**). During fetal life, primordial germ cells (PGCs) will develop into oocytes and stay arrested until the later recruitment to form the preovulatory follicles. At this stage, the oocytes will be lined by two types of granulosa cells, which are mural granulosa cells on the inner surface of the follicle and the cumulus cells on the outer surface, closer to the gametes (3). For the preparation of ovulation, the cumulus cells will go through the process of ‘cumulus expansion’ whereby hyaluronic acid is produced and deposited into the extracellular space together with other proteins. The oocyte on the other hand, will resumes meiotic division and form a mature cumulus-oocyte complex (COC). This complex contains an oocyte arrested at metaphase of the second meiotic division (MII) and is ready for ovulation and subsequent fertilization (4). The follicular development and ovulation are much affected by the communication of the oocytes with the surrounding granulosa cells as well as the conducive state of the follicular microenvironment.

In PCOS, the impaired folliculogenesis started with the hyperandrogenic state (5). The excess free testosterone will increase the release of high pulse frequency of GnRH from the hypothalamus as well as gonadotropin hormone from the pituitary. In addition, the luteinizing hormone will promote androgen production in ovarian theca cells, while the follicular stimulating hormone act on the ovarian granulosa cells to transform the androgens into estrogens. This imbalance of the hypothalamic-pituitary-ovarian axis will lead to an excessive ratio in PCOS women (6). Consequently, ovarian function is impaired and the developing follicles ceased at an early stage leading to the abundance of small immature follicles. Over-production of anti-Mullerian hormone (AMH) by the small follicles inhibits FSH-induced aromatase activity and hinders follicular development which further results in ovarian malfunction (5). These underdeveloped follicles are the basis to the name of PCOS. The morphology of the growing follicles is heterogeneous in number, size, and maturity. These excess follicles however did not represent good quality follicles as they were not fully developed and attained the full potential to become a competent oocyte. Thus aggravating the follicular arrest (5, 6). Hence, molecular markers of granulosa cells are a potentially reliable tool for assessing oocyte quality (7).

In folliculogenesis, the ultimate final step is the expulsion of mature oocytes following the tissue remodeling process. Previously it was hypothesized that the follicular rupture could be due to an increase in intrafollicular pressure. However, recent findings proved that inflammation *via* gonadotropin stimulation plays a key physiologic role in this process. The inflammatory process includes direct and indirect action to cause vasodilation, hyperemia, edema, collagenolysis, cell proliferation, and eventually resulted in the weakening of the follicle wall and rupture (8). Inflammation may produce oxidative stress and vice versa. A study on PCOS women demonstrates that there is an increase in oxidative stress in addition to a decreased antioxidant capacity (9). The by-product of oxidation which is reactive oxidative species (ROS) is important for ovulation but will be harmful to the cells if produced in excess. The resulting ROS will trigger a chain reaction of the inflammatory markers namely nuclear factor-κB and TNFα, which subsequently promotes insulin resistance. Besides, there is an upregulation of IL8, IL1B, nitric oxide synthase 2 (NOS2), and prostaglandin-endoperoxide synthase 2 (PTGS2) in granulosa cells that can impair maturation and subsequent ovulation (10). It can also contribute to a higher risk of oocyte aneuploidy by disrupting the mitochondrial morphology and function resulting in abnormal chromosome arrangement. Both chronic inflammation and ROS generation can alter the normal ovarian follicular dynamics resulting in poor oocyte quality, a higher risk of miscarriage, and reduced women’s reproductive potential in PCOS (6).

## Oocyte Assessment Strategies

One of the most important steps in human-assisted reproduction is embryo selection and transfer. Usually, not all harvested oocytes are transferred due to the risk of multiple gestations, thus one had to evaluate the embryos’ quality and select the best with the highest pregnancy probability. This is even more challenging due to the short interval for the assessment. Thus, it is crucial to develop an objective and accurate test to assess the quality of oocytes and embryo viability.

**Figure 2:**
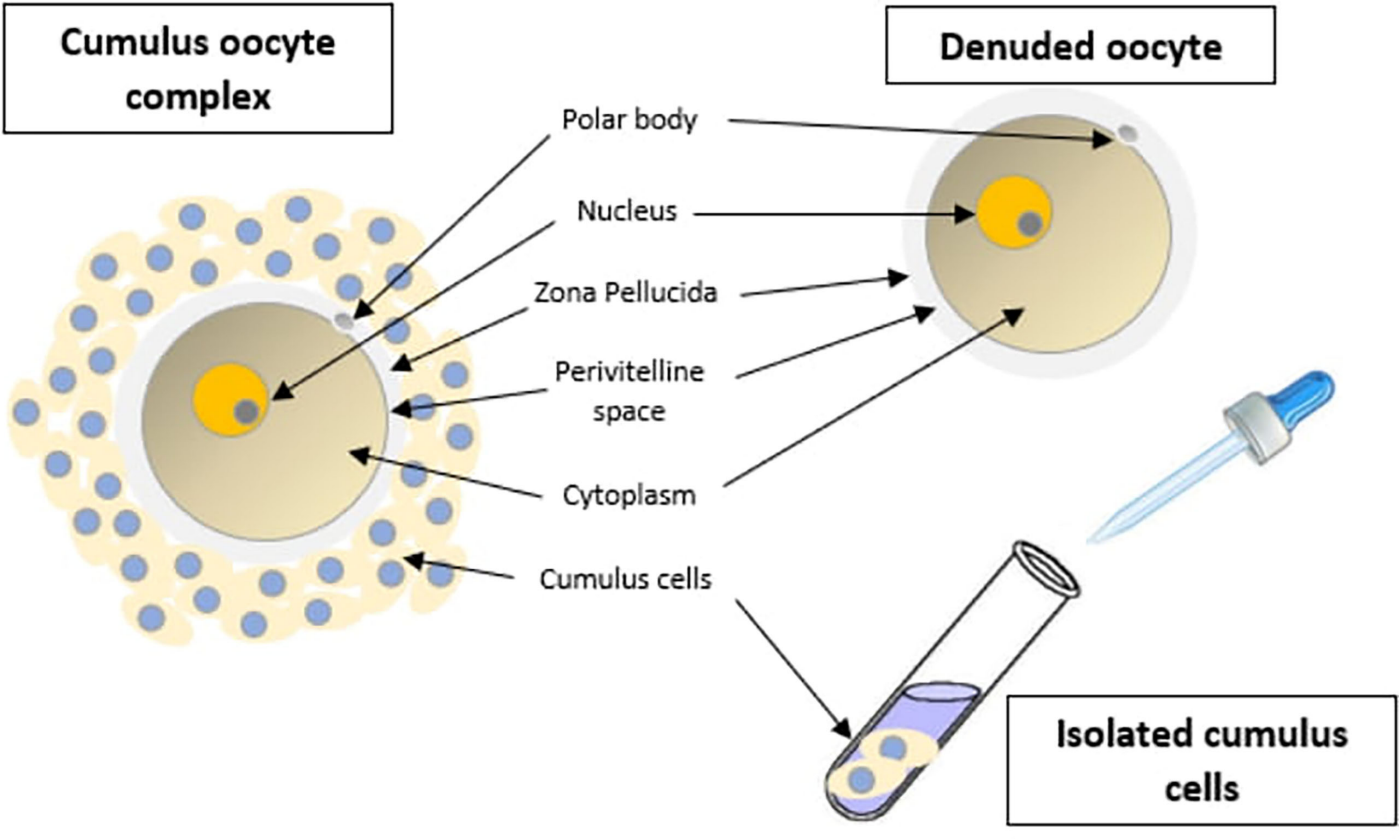
Close-up diagram of the cumulus-oocyte complex.

Currently, embryo morphology and developmental criteria during *in vitro* development are the most common non-invasive assessment strategies used (11). However, this assessment has limitations and still has room for improvement. The oocyte which is separated from the cumulus cell oocyte complex will be evaluated based on the nuclear maturation status, the morphology of the cytoplasm, and the appearance of the extra-cytoplasmic structures (**Figure 2**). Each criterion will be given a score of -1 (worst), average (0), or best (1) and total oocyte scores will be assigned by adding up the individual parameter score (**Table 1**). Each criterion symbiotically contributes to the high fertilization rate. For example, a good mature nucleus alone is insufficient to determine the top quality of the oocyte. It also needs to have a good perivitelline space (PVS) or a granular cytoplasm to have a better chance to be fertilized. However, these MII phase oocytes are very fragile and susceptible to damage from all the repeated assessment processes. There are also other methods of morphological assessment i.e. cleavage-stage morphology scoring on day 2 after fertilization (12), weak or low-grade embryo (WLGE) (13), score on the expansion of the blastocoel cavity, the cohesiveness of the inner cell mass, and the trophectodermal cells (14).

**Table 1:** The figure represents the oocyte grading system for the six morphological characteristics analyzed.

Criteria	-1 (poor)	0 (slight poor)	1 (normal)
Oocyte morphology	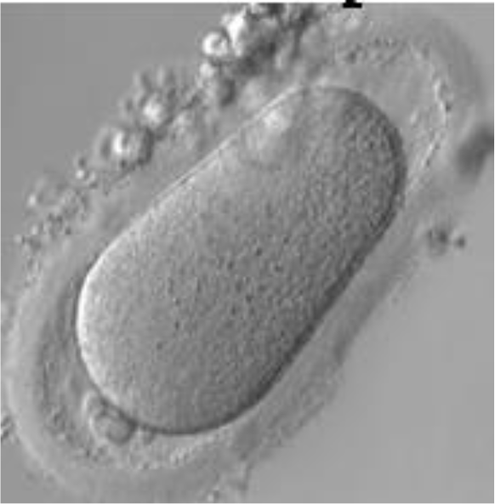 Dark general oocyte color and/or ovoid shape	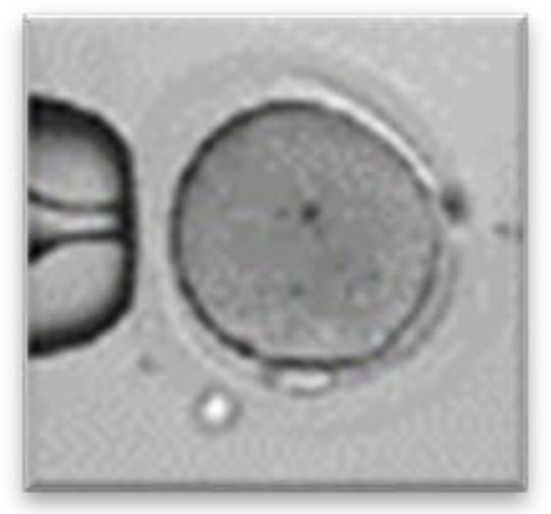 Less dark general oocyte color and less ovoid shape	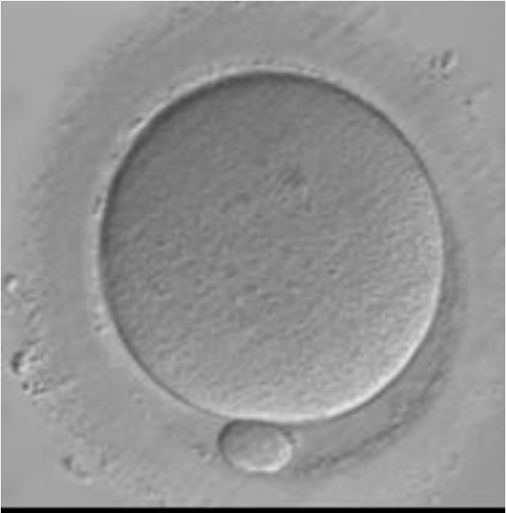 Appeared normal
Oocyte size	The size was less than 120μ or greater than 160μ.	Did not deviate from normal by more than 10μ.	Within normal range > 130μ and <150μ.
Oocyte cytoplasm	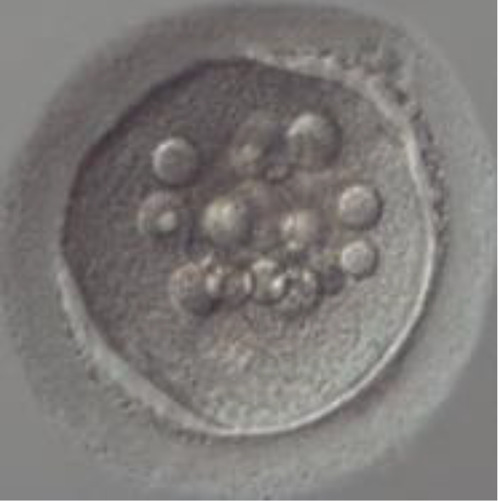 The cytoplasm was very granular and/or very vacuolated and/or several inclusion bodies.	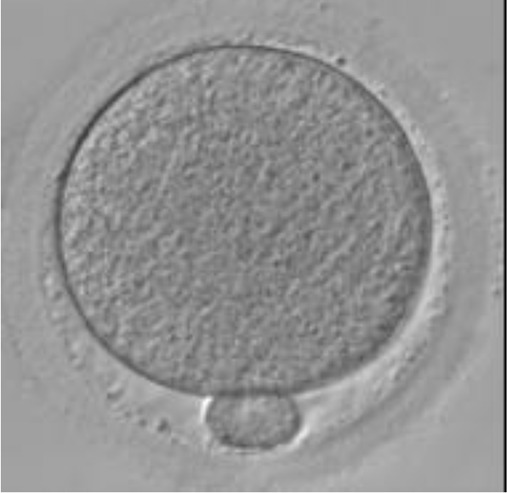 Slightly granular and/or only a few inclusion bodies.	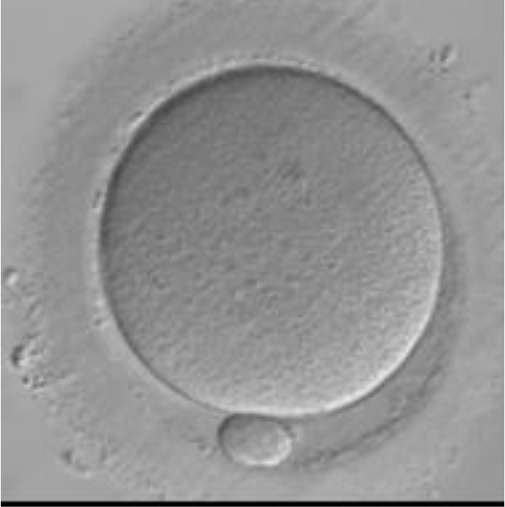 Absence of granularity and inclusion bodies.
Oocyte zona pellucida	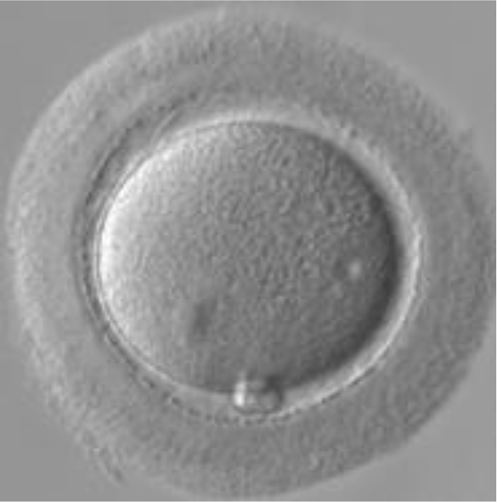 Very thin or thick (<10μ or >20μ)	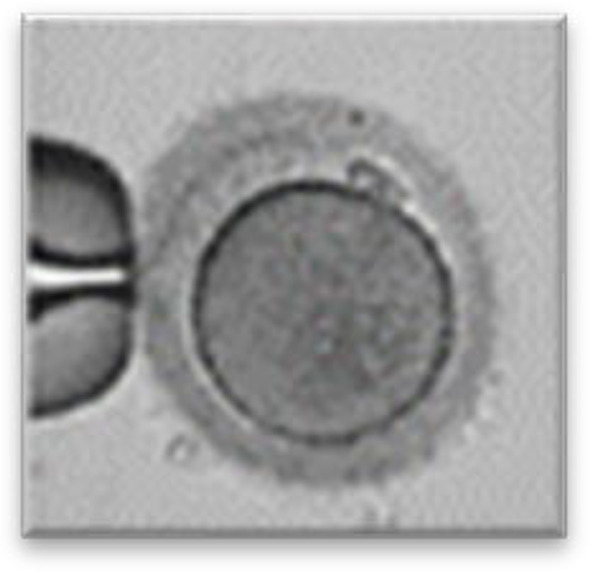 Did not deviate from normal by more than 2μ	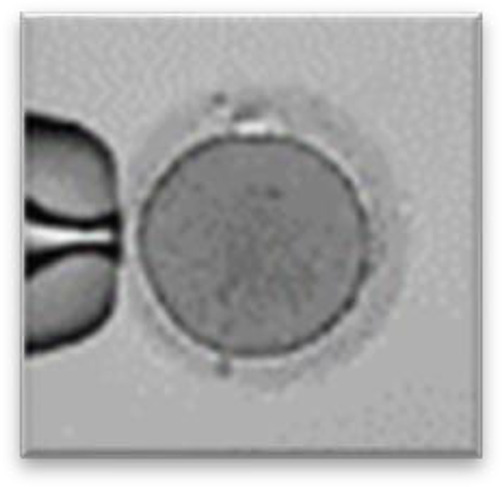 Normal zona (> 12 μ and <18 μ)
Oocyte pervitelline space	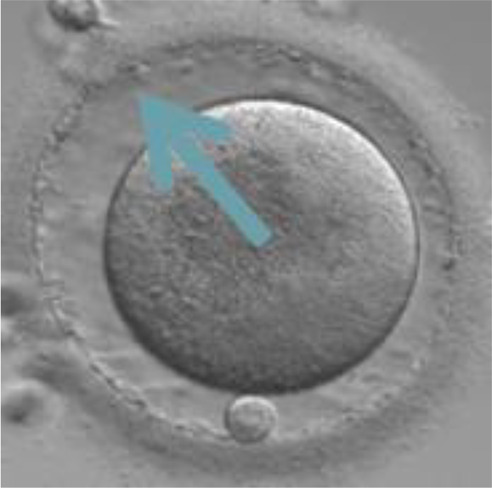 Abnormally large PVS, an absent PVS,	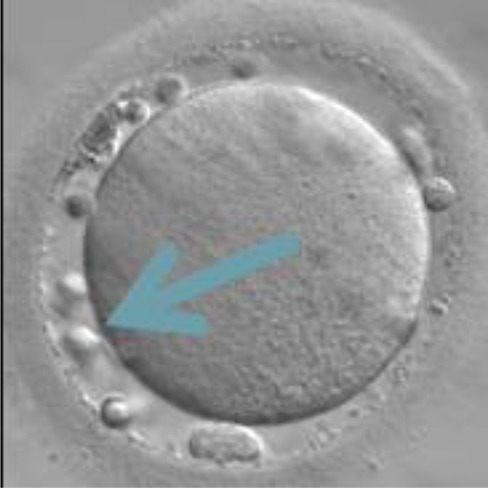 Moderately enlarged PVS and/or small PVS	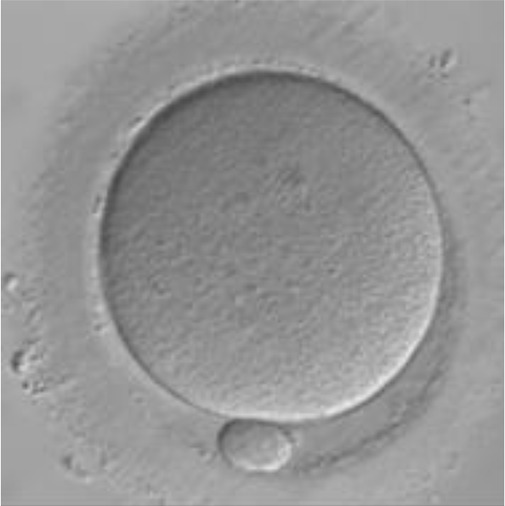 Normal size PVS with no granules
Oocyte polar body	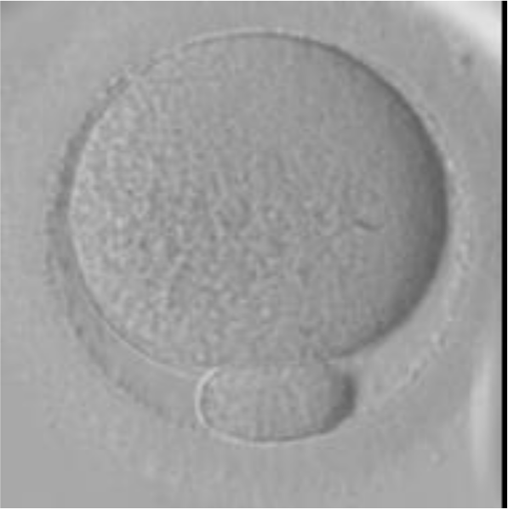 Flat and/or multiple PBs, granular and/or abnormally small or large PBs	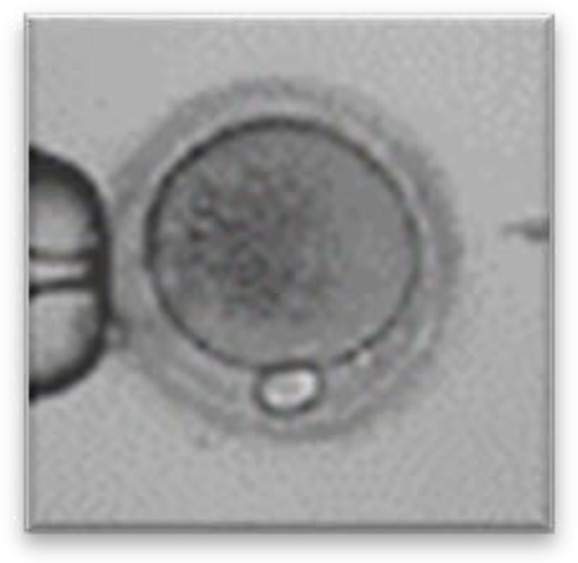 Fair but not excellent	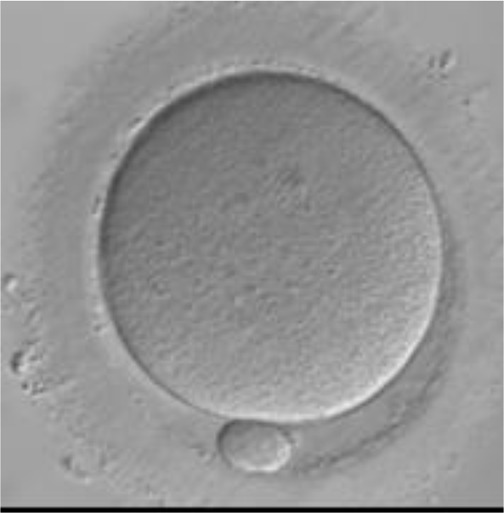 Normal size and shape

Every single characteristic was graded as worst (-1), average (0), or best (1) creating a total oocytes scores.

Besides all these extensive assessments, a surprising finding by Munné at. al reported that most of these morphologically normal-appearing embryos (based on shapes, sizes, blastomere numbers, and fragmentation) were possibly aneuploid. The percentage of morphologically good, aneuploid embryos also increased significantly from 56% in women aged less than 35 years old to nearly 80% in women aged 41 and older (15). These data emphasize the urgent indication to improve the current assessment method, hence improving the ART success rate. Consequently, recent studies start to evaluate oocyte quality based on other methods, including the study of epigenomics, transcriptomic analysis of the cumulus/granulosa cells, and proteomic and metabolomic analysis of embryo culture media (16–18).

## Gene Expression in the Cumulus Cells

The analysis of cumulus cells provides good morphological and metabolic information about the oocytes and complements the current assessment method. The cumulus cell has a reciprocal functional interconnection with the oocyte (2, 18, 19). During oocyte maturation, the cumulus cells provide channels to allow for the transport of nutrients, regulatory molecules, and paracrine factors. Transportation of these essentials will promote nuclear and cytoplasmic maturation of the oocyte, hence acquiring developmental competence (20). Conversely, oocytes will secrete factors to promote cumulus cell differentiation and expansion. Cumulus expansion is critical for normal oocyte development, ovulation, and fertilization and those matured oocytes without cumulus expansion have limited potential for implantation (21). Therefore, gene expression levels in the cumulus cells act as a mirror of the true oocyte developmental potential (13, 16, 22). (**Table 2**) summarize the cumulus gene expression according to their physiological function and its association with the quality of embryo. To date, there is no single gene expression that can predict which embryos produced by ART will be implanted and lead to a live birth (17). However, ART may improve the outcome, especially in PCOS women as evidenced by normalization of the expression of genes participating in vital processes such as cell proliferation, hormone receptors signaling, gap communication, folliculogenesis, and oxidative stress (39). Other studies also demonstrated that PCOS women had improved fertilization, cleavage, implantation, clinical pregnancy, and live birth rate following ART (14, 39, 45–49, 82), while others showed no difference (50) or worse than control (51). Another study reported a higher number of oocytes retrieved from PCOS patients, but the quality of oocytes was not statistically different (52). It can be concluded that the cumulus gene expression may have a diagnostic and therapeutic role for PCOS patients to overcome infertility.

**Table 2:** Cumulus gene expression according to the physiological function and its correlation with the quality of embryo.

Function	Genes involved	Expression level	Studies
Cumulus expansion	HAS2	High in high-quality blastocyst	Kahraman (23)
		High in the high-grade embryos	Gebhardt (12)
		High in good quality MII oocytes (P<0.05)	Ekart (24)
		High in the high-quality embryo (P<0.05)	Cillo (21)
		High in oocytes with high developmental potential	Assidi (25)
		High in the high-quality embryos (P<0.001)	McKenzie (26)
		Low expression in PCOS	Patil (27)
	GREM1	-Positive correlation with a good embryo or blastocyst morphology (P<0.01)	Wathlet (22)
		-Possible tool for developmental potential, but no significant differences between groups resulting in pregnancy with live birth or not. -Positive correlation with birth weight at term (P≤0.08) -Low expression in high grade embryos	Gebhardt (12)
		-High in fast-developing embryos (embryos with ≥7 cells) (P<0.05) -Expression related to gonadotrophin preparation (P<0.05) -High in older age group (P<0.05)	Adriaenssens (13)
		High in oocytes with high developmental potential	Assidi (25)
		High in the high-quality embryo (P<0.05)	Cillo (21)
		High in the high-quality embryos (P<0.001)	McKenzie (26)
	VCAN	-High in the high-quality embryos (P=0.024) -Lower in implanted embryos group (P<0.05)	Shen (20)
		No significant difference between cumulus cells that developed into implanted vs nonimplanted embryos	Burnik (28)
		High in good quality MII oocytes (P<0.05) and oocytes resulted in a healthy term live birth outcome (P<0.05)	Ekart (24)
		-High in cumulus cells from oocytes yielding pregnancy resulting in a live birth (P=0.02). -Positive correlation with birth weight at term (P≤0.08). -High in the high-grade embryos	Gebhardt (12)
		-Low in mature oocytes -Good pregnancy predictor, the sensitivity of >70% and specificity >90%	Wathlet (22)
		-High in the mature oocyte (P<0.005) -Expression related to gonadotrophin preparation (P<0.05)	Adriaenssens (13)
		Low expression in insulin resistance PCOS group	Hassani (29)
	PTGS2	-No significant difference	Burnik (19)
		-High in cumulus cells from oocytes yielding pregnancy resulting in a live birth (P=0.02). -Low in the high-grade embryos	Gebhardt (12)
		-High in mature oocytes and good quality embryo (P<0.01)	Wathlet (22)
		-The expression was inversely correlated with the embryo development stage on Day 3 -The expression is affected by the types of gonadotrophin treatment	Adriaenssens (13)
		High in the oocyte with good development potential	Assidi (25)
		High in the high-quality embryos (P<0.05)	McKenzie (26)
		High in PCOS	Schimdt (10)
	TNFAIP6	Low in the fertilized oocyte (P=0.044)	Shen (20)
		-No significant differences between groups resulting in pregnancy with live birth or not. -High in the high-grade embryos	Gebhardt (12)
		High expression in cumulus cells resulted in pregnancy (P>0.05)	Hamel (18)
		High in good quality embryo	Assidi (25)
		No differences were observed in embryo quality	McKenzie (26)
		Low expression in PCOS women which resulted in infertility	Patil (27)
	PTX3	High in cumulus cells resulted in pregnancy	Feuerstein (30)
		-High in cumulus cells from oocytes yielding pregnancy resulting in a live birth (P=0.06). -High in the high-grade embryos	Gebhardt (12)
		No differences were observed in embryo quality	Cillo (21)
		High in cumulus cells that developed into embryos with high implantation potential	Zhang (31)
		Gene was not detected	McKenzie (26)
		High in PCOS	Pan (32)
	SDC4	-Positive correlation with a good embryo or blastocyst morphology (P<0.01) -Good pregnancy predictor, the sensitivity of >70% and specificity >90%.	Wathlet (22)
		-High pregnancy prediction	Kordus (17)
		-No differences were observed in embryo quality -Expression related to gonadotrophin preparation (P<0.05)	Adriaenssens (13)
	STS	No significant differences between groups resulting in pregnancy with live birth or not	Gebhardt (12)
	AHR	No significant differences between groups resulting in pregnancy with live birth or not	Gebhardt (12)
Cumulus apoptosis	Caspase family	Low in high-grade embryos	Brentnall (33)
		High in PCOS	Salehi (34)
	Survivin	High in high-grade embryos	Brentnal (33)
		Low in PCOS	Salehi (35)
	Bcl-2	High expression in the fertilized egg	Dehghan (36)
		High in PCOS	Mikaeli (37) Honnma (38)
		No significant difference between PCOS and control	
	SOD1	Increase expression in increased oocyte competency and embryo development	Da Broi (2)
		Increased in PCOS	Liu (39)
Glucose metabolism	PFKP	Higher in mature oocytes (P=0.014)	Shen (20)
		High prediction of good blastocyst formation	Hammond (40)
		-Possible assessment tool, but no significant differences between groups of pregnancy with live birth and not. -High in the high-grade embryo -Positive correlation with birth weight at term (P<0.05).	Gebhardt (12)
		Low expression in PCOS women which may relate to low competency oocytes	Zhai (41)
	PKM2	-Lower in implanted embryos (P<0.05) -Higher in mature oocytes (P<0.05)	Shen (20)
		High in oocytes with developmental potential	Gebhardt (12)
		Low expression in PCOS	Liang (42)
	LDHA	The expression level was not associated with oocyte maturity, fertilization, embryo grade, and implantation (P>0.05)	Shen (20)
		High predictive of blastocyst formation	Hammond (40)
		-Possible assessment tool, but no significant differences between groups resulting in pregnancy with live birth or not. -High in the high-grade embryos	Gebhardt (12)
		Low expression in PCOS which may relate to low competency oocytes	Liang (42) Zhai (41)
	GFPT1,2	The expression level was not associated with oocyte maturity, fertilization, embryo grade, and implantation (P>0.05)	Shen (20)
	G6PD	Silencing of the gene in cumulus cells significantly impaired oocytes maturation.	Xie (67), Wen (44)
	SLC2A4 (also known as GLUT4)	No significant difference in high-quality MII oocytes	Ekart (24)
		Positive correlation with cleavage rate and developing embryo	Hammond (40)
	ALDOA	High in oocytes with high developmental potential	Gebhardt (12)

Phosphofructokinase (PFKP), Pyruvate kinase (PKM), Lactate dehydrogenase (LDHA), Glutamine--Fructose-6-Phosphate Transaminase (GFPT), glucose-6-phosphate dehydrogenase (G6PD), Solute carrier (SLC), Hyaluronic synthase 2 (HAS2), Gremlin1 (GREM1), Versican (VCAN), Prostaglandin synthase 2 (PTGS2), Tumour necrosis factor α-induced protein 6 (TNFAIP6), Pentraxin 3 (PTX3), Syndecan-4 (SDC4), Forehead BoxO 3(FOXO3), B-cell lymphoma 2(Bcl-2), Fatty acid synthase (FAS) and Superoxide dismutase 1(SOD1).

The first potential markers for determining oocyte quality are the member of growth factor superfamily, growth differentiation factor 9 (GDF-9), and its homologous bone morphogenetic protein 15 (BMP-15 or GDF-9B). Both are secreted by the oocytes and act as potent regulators for primordial follicle recruitment (53), proliferation, differentiation, and expansion of granulosa cells (54), and steroid synthesis (55). GDF-9 and BMP-15 initiate signaling pathways by binding to type I and type II receptors, hence activating Smad 2/3 pathway and Smad 1/5/8 pathway respectively (56). Another study on mice showed a complete loss of GDF-9 in homozygous males does not affect fertility in contrast to homozygous null female that were infertile (83). They also demonstrated that GDF-9-deficient oocytes may grow normally at the early stage but arrested at the late secretory events (83). Mice deficient in this type 1 receptor (BMPR1B) exhibited irregular estrous cycles and impaired oocytes with defected cumulus expansion which resulted in low fertilization (56) Another study on mice showed a complete loss of GDF-9 in homozygous males does not affect fertility in contrast to homozygous null female that were infertile (83). They also demonstrated that GDF-9-deficient oocytes may grow normally at the early stage but arrested at the late secretory events (83). In human follicles, BMP-15, GDF-9, and its receptors were expressed in all stages of follicles development and corpus luteum (57). A study by Yoshino showed higher BMP-15 in oocytes of mature follicles and regulated cumulus expansion in mice (58). Hence, GDF-9 and BMP-15 are important determinants for ovulation, fertilization, and embryonic competence.

Many researchers had specifically evaluated the expression levels of GDF9 downstream target genes in the cumulus cells such as hyaluronic synthase 2 (HAS2), pentraxin 3 (PTX3), gremlin1 (GREM1), prostaglandin synthase 2 (PTGS2), and versican (VCAN). For example, cumulus cells with high expression of PTGS2, VCAN, HAS2, and GREM1 were shown to produce higher quality embryos than those with low expression levels (16, 21, 25, 26, 31). BMP15 on the other hand helps to provide energy substrates by enhancing glycolytic activity and expression of glycolytic enzymes in cumulus cells to meet the high demands of oocytes’ energy requirement (20). These glycolytic enzymes include phosphofructokinase (PFK), pyruvate kinase (PKM2), and lactic dehydrogenase (LDHA). The energy-producing mechanism is essential for oocyte development and maturation.

There were different findings in studies comparing these two critical oocyte factors in PCOS women. Some studies showed delayed and downregulation of GDF-9 and BMP-15 mRNA expression in cumulus cells of PCOS women which may result in a higher risk of miscarriage due to premature luteinization, poor oocyte quality, and luteal dysfunction (59, 60). Treatment of the COCs with recombinant GDF-9 and BMP-15 in ART following IVM resulted in higher rates of blastocyst formation and better fetal yield in cattle, mice, goats, and pigs (60). Another study showed a significantly lower level of GDF-9, with no change in BMP-15 among PCOS women when compared to control (61, 83). This is probably due to the specific role of GDF-9 in maintaining the follicle structure which can contribute to the long-term developmental potential of the oocytes.

The complex genetic etiology of PCOS requires studies to compare the level of gene expression at multiple stages during oocytes and cumulus cell development. Besides, one of the studies showed the presence of a list of cumulus cells genes in mature PCOS oocytes which were not found in the previous studies of normal oocytes. They are RUNX2, PSAT1, ADAMTS9, CXCL1, CXCL2, CXCL3, and ITGB5 (14, 49). This proves the possibility of different molecular mechanisms involved in the process of oocyte nuclear maturation in PCOS compared to non-PCOS.

### Genes Related to Cumulus Expansion and Apoptosis

**Figure 3:**
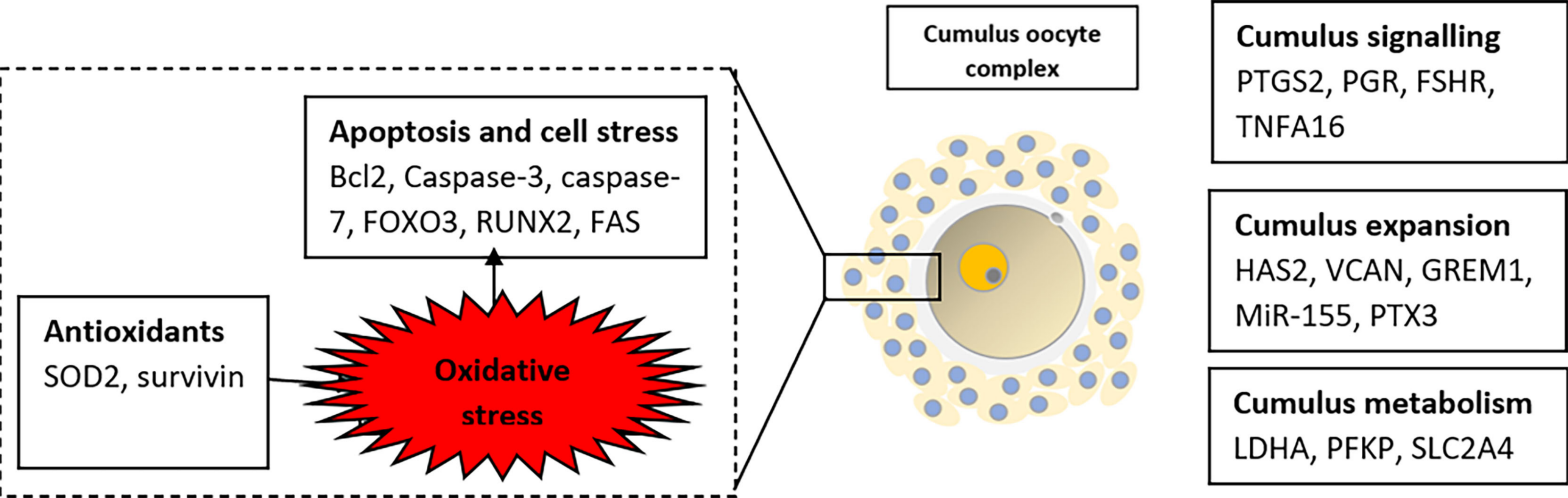
Summary of selected potential genes related to embryo viability in human cumulus cells.

During folliculogenesis, the cumulus cells will undergo the process of “cumulus expansion”, whereby a new extracellular matrix (ECM) will be formed to bind the oocyte and cumulus cells together, hence enabling the oocyte to resume maturation. The extracellular matrix of COC is composed of several molecules with varying roles such as differentiation, division, cell apoptosis, and migration (**Figure 3**). Successful fertilization is sensitive to any changes to the composition and functional capacity of the ECM.

Hyaluronic synthase 2 or also known as HAS2 is responsible for the production of one of the main components of the extracellular matrix, the hyaluronan. FSH, LH, and GDF9 combinedly affect the expression of HAS2, especially at cumulus cells. Studies showed higher expression of HAS2 in the high-quality embryo (21, 23, 25) while McKenzie proved that HAS2 expression in cumulus yielding higher grade embryos was 6-fold higher than those from lower grade embryos. However, contradicting result was reported by Shen et al. (20), where the expression of HAS2 was not associated with the oocyte quality and implantation rate. Besides the HAS2 gene, PTX3 also has a clear role in cumulus expansion. This molecule co-localizes with hyaluronic acid throughout the cumulus matrix with the help of tumor necrosis factor α-induced protein 6 (TNFAIP6) and is essential for *in vivo* fertilization (32). Inactivation of this gene reduced the ability of oocytes to fertilize, by disrupting the structural integrity of the cumulus oocytes complex. Zhang et al. found a relative abundance of PTX3 mRNA in cumulus cells from oocytes that developed into embryos with higher implantation potential. He also noted the level of PTX3 expression was 12 times greater than those from oocytes that did not fertilize (31). This is further evidenced by a study on PTX3 knockout female mice that appeared to be grossly normal, but clinically sub-fertile (62, 63). In contrast, the studies by McKenzie et al. (26), Cillo et al. (21), and Shen et al. (20) failed to prove the significant association. Nonetheless, other studies expressed positive relation between PTX3 and pregnancy outcome (12, 30).

In PCOS, the expression of TNFAIP6 and HAS2 was significantly reduced in human cumulus granulosa cells (27). This potentially resulted in altered structural constituents and intrinsic defects in granulosa cells and may contribute to infertility. In the same study, they noted that only HAS2 expression was restored upon treatment with growth factor supplementation: amphiregulin (AREG) and GDF-9, while TNFAIP6 remains the same. With regards to PTX3, recent research demonstrated that PTX3 in circulation is associated with PCOS (64, 65) but its role is so far inconclusive. There was a significantly higher level in the PCOS women than in control which supports the low-grade inflammatory state in PCOS (32). In contrast, another study found no significant difference in the level of PTX3 between cumulus cells in the MII and MI/GV stage, however, there were significant differences in PTX3 level between cumulus cells isolated from mature oocytes that formed two normal pronuclei or multi-pronuclei after fertilization (14).

Another gene that favors cumulus expansion is GREM1 expression. Inactivation of this gene resulted in reduce fertilization rate (14). A study in mice by Pangas et al. found out that GREM1 is possibly involved in the communication between GDF9 and bone morphogenetic protein (BMP) signaling, on the basis that they are sharing the same ligands of the same signaling pathway. This communication is important for follicular development thus can be related to oocyte quality. Glister et al. (84) showed that the relation between GREM1 and intrafollicular BMP contributes to the negative regulation of thecal androgen production while Cillo reported high expression of GREM1 in high-quality oocytes (21). There is an abnormal expression of GREM1 in the early oocyte development stage with subsequently normalized level after treatment among PCOS women undergoing ART (39).

The next gene of interest is versican protein. It is a component of the extracellular matrix that stabilizes hyaluronan. It is involved in cell adhesion, proliferation, migration, and angiogenesis (28). Versican expression is closely regulated by three main hormones: FSH, LH, and HCG. It has been shown that *in-vitro* matured mouse cumulus-oocyte complexes mice injected with HCG resulted in significantly down-regulated transcription and translation of the versican gene in follicles (66). Besides, VCAN also has EGF-like effects on gene expression in the cumulus-oocyte complex, which is very important for the final maturation of oocytes. This is proven when versican protein was supplied into media for *in vitro* mouse oocyte maturation, cumulus-specific genes like PTGS2, TNFAIP6, and HAS2 become more significant (66). Interestingly, there were contradicting results for the relationship between VCAN cumulus cells expression and oocyte developmental competence. One group of studies showed gene expression of VCAN positively correlated to day 3 embryo grade (13, 20) but did not correlate with implantation competence after adjusted to age and BMI (20, 21, 26). On the other hand, other studies reported that VCAN expression was higher in good-quality blastocysts, among the live birth group (12, 24) and pregnancy group, while others showed no difference (19). A comparison study between the PCOS insulin resistance group and insulin-sensitive group demonstrated the downregulation of VCAN genes. However, no significant changes were noted when comparing all PCOS patients to control (67).

The variance in all these genes expression is possibly due to different materials and methods used in the studies. Different types of cumulus cells samples (pooled vs non-pool) will yield different results. Besides, Wathlet et al. pointed out that there is a wide variation for almost all gene expression levels even within the cumulus cells of the same individual (22). Other factors like sample size also play a role. Most of these studies were conducted in small sample size (12, 21, 24, 26, 31), thus predisposed them to sample bias, like age, BMI (20, 68), clinical characteristics and IVF protocols used (13, 22).

Besides the above-mentioned genes, there are other genes expressed in n the extracellular matrix of the COC among PCOS women which involved in the composition and regulation of ECM during the ovulation process. Compared to the control, 21 genes were expressed differently from which 18 are downregulated. The genes are extracellular matrix protein 1 (ECM1), catenin (cadherin-associated protein), alpha 1 (CTNNA1); integrin, alpha 5 (ITGA5); laminin, alpha 3 (LAMA3); laminin, beta 1 (LAMB1); fibronectin 1 (FN1); and integrin, alpha 7 (ITGA7) (67). Laminin and fibronectin formed the basement membrane that surrounds the granulosa layers of all follicles. All these data show that the genes are not isolated, independent genes, but formed interactions with their produced proteins and cooperate as a cluster.

A great balance between cumulus expansion and apoptosis is crucial to achieving successful ovulation. Deterrent apoptosis of cumulus cells leads to poor oocyte outcomes, embryo fragmentation, and impaired blastocyst development (2, 35). The percentage of apoptotic cells in women who achieved pregnancy was significantly lower than those who failed to achieve pregnancy (34). Apoptosis is an active process of cellular deconstruction triggered by certain conditions or stimuli. In a normal functioning ovary, apoptosis is important for follicular development *via* atresia and luteolysis, as well as embryo implantation *via* decidualization and placentation. The molecular basis of apoptosis initiation in the ovary is not fully understood. Among the molecules that are known to regulate apoptosis are members of the Bcl-2, Caspase family, anti-Mullerian hormone (AMH), and survival factors such as inhibitors of apoptosis protein (IAP) family, as well as the gonadotrophins (LH and FSH). Besides that, cumulus cells also provide protection against pro-apoptotic cells. For example, SOD1 in the cumulus cells provide antioxidant defences that protect the oocyte from ROS and oxidative stress-induced apoptosis. A study in infertile patients with stage III/IV endometriosis who achieved clinical pregnancy after IVF showed higher SOD1 expression compared to a milder case (2), suggesting SOD1 may protect the oocyte from oxidative damage.

Survivin is the smallest member of the IAP gene family, which is involved in the embryonic development and proliferation of normal adult tissues such as skin, endometrium, and granulosa cells (69). Survivin regulates cell apoptosis by direct or indirect inhibition of caspase-3 and -7 which eventually lead to events promoting cell suicide. Caspase-3 act as the main executor, causing chromatin condensation, DNA fragmentation, nuclear collapse, plasma membrane blebbing, and processes associated with cell disassembly and apoptotic body formation. While caspase-7 plays a supportive role in the demolition phase by increasing the production of reactive oxygen species (ROS) and separating the cells from the ECM (33).

The gene expression of these apoptotic regulators was found to be altered in PCOS women resulting in poor-quality embryos (35, 70). One of the possible hypotheses suggested that the high rates of apoptosis in cumulus cells diminish the physical and nutritional support that the cumulus cells offered to the COCs (35). Consequently, low-quality oocytes were produced, resulting in poor fertilization, and reduced embryo development (71, 72). One study demonstrated that for every one percent increment in CC apoptosis, the clinical pregnancy and live birth rates will be decreased by 11-12% (73). There are many external as well as internal stimuli that promote this alteration, such as endocrine abnormalities, high levels of stress oxidative products, hyperandrogenic state, and imbalance of the LH/FSH ratio.

In cumulus cells of women with PCOS, the survivin (antiapoptotic) gene expression was lower while the other pro-apoptotic genes namely caspase-3, caspase-7, FOXO3, RUNX2, FAS cell surface death receptor were highly expressed compared to control (35, 37–39). FOXO3 is an intrinsic regulator of apoptosis which acts by upregulating the FAS ligand and BCL 211 (37). Fas (APO-1, CD95) is a member of the tumor necrosis factor (TNF) which can signal a cell to undergo apoptosis *via* activation of caspases. In PCOS, both Fas and its ligand, Fas ligand were highly expressed in ovarian vascular myocytes but showed no difference in normal ovaries (74). This may suggest that FAS-mediated apoptosis does not directly contribute to the disordered folliculogenesis of PCOS but rather causes impaired oocyte function *via* ovarian vascular remodeling.

B-cell lymphoma 2 (Bcl2) families of proteins are regulators of cell death and survival. The expression is increased in cumulus cells of matured and fertilized oocytes (36). Altered expression of these genes in cumulus cells and oocytes affects oocyte development in PCOS (59). This proved that Bcl2 expression increased the ability of oocytes to complete nuclear maturation and become fertilized. In addition, Bcl2 also acts as another target gene for MiR-155. Increased miR-155 expression resulted in the downregulation of Bcl2 (36). MicroRNA MiR-155 is involved in promoting cumulus expansion, nuclear and cytoplasmic maturation, as well as cleavage process. MiR-155 is overexpressed in granulosa cells of PCOS and negatively affects nuclear and cytoplasmic maturation but has a positive impact on embryo development (36).

RUNX2 is a transcription factor involved in cell differentiation. The expression of RUNX2 is negatively related to embryo development potential and can be used as a genetic biomarker for the assessment of embryo quality (14). RUNX2 is not an apoptotic cell, but the mechanism of action is similar to other apoptosis-related genes. Under the hypoxic condition, it will induce the formation of ROS, leading to lipid peroxidation, enzyme inactivation, and cell damage which finally resulted in apoptosis (75).

Data regarding the apoptotic gene expression in PCOS were controversial. In contrast, reports were showing a low or normal level of apoptotic biomarkers in cumulus cells of PCOS women (76) and even no proof of the correlation between cumulus cells apoptosis and oocyte maturity (77). Little is known regarding the molecular roles of apoptosis-related genes in the development of oocyte and embryo qualities in PCOS women, hence more studies are needed to analyze this potential biomarker.

### Genes Related to Glucose Metabolism

Like other cells in the body, glucose is an essential metabolite for the cumulus cell oocyte complex. It can be utilized for energy production, cellular homeostasis, nuclear maturation, substrates for matrices production, and signaling. Due to the poor capacity of glucose uptake by the oocyte itself, the role of cumulus cells is paramount to help the process and supply metabolic intermediates that are readily utilized by the oocyte. This is proven from the findings in which the cumulus cells from immature COCs consume 23-fold more glucose and 3.2-fold less oxygen than oocytes; compared to mature bovine COCs which consume only two-fold more glucose, oxygen, and pyruvate (78).

**Figure 4:**
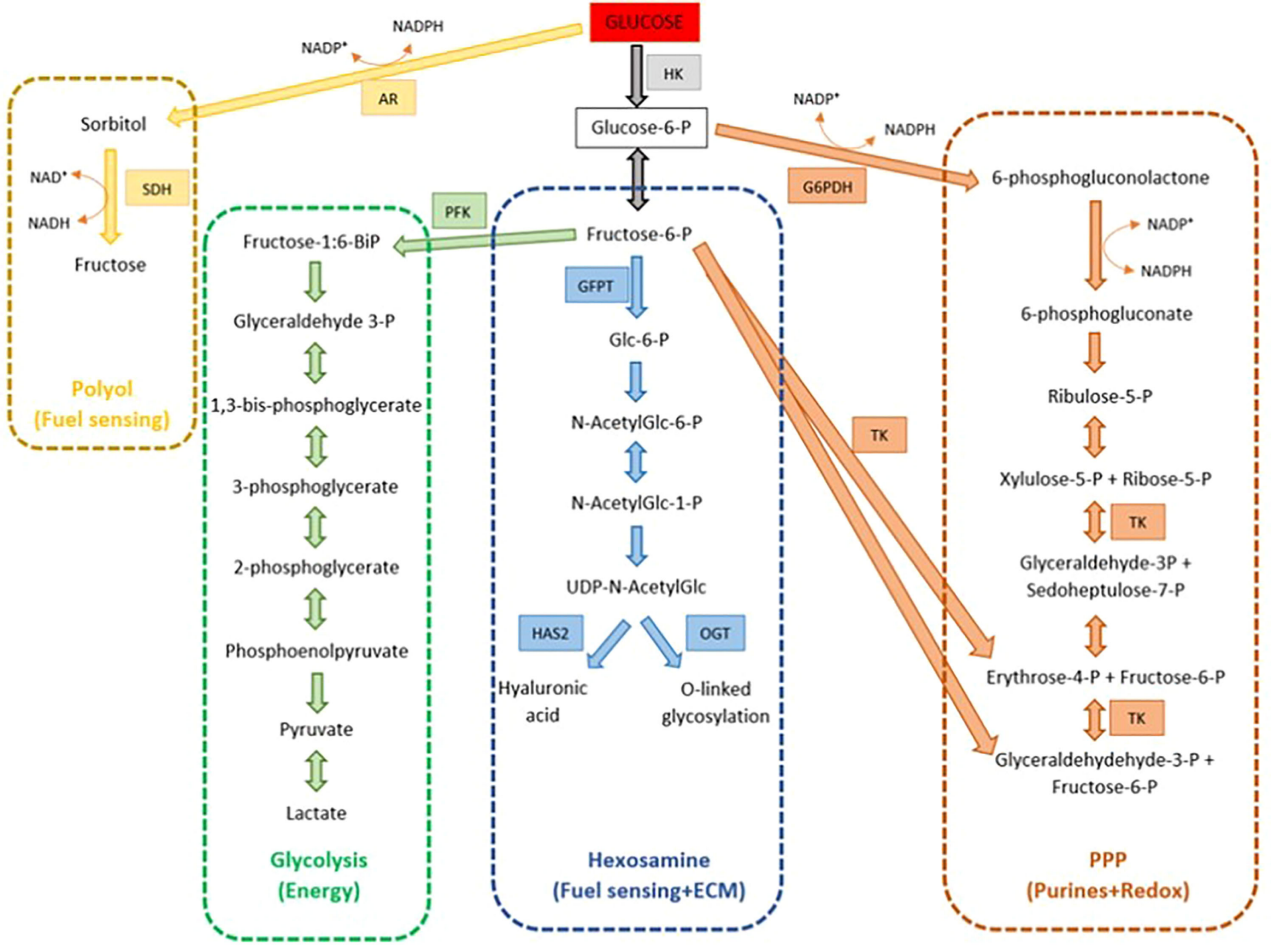
Multiple glucose metabolism pathways within the cumulus-oocyte complex (COC).

The glycolytic pathway is the most predominant pathway for glucose metabolism (**Figure 4**). It acts mostly as an energy supplier for cytoplasmic expansion. As a result, small molecule energy metabolites such as pyruvate and lactate are produced and can be readily transferred and used by the oocytes. The high capacity of cumulus cells to metabolize glucose depends on the presence of glucose transporters (SLC2A1 and SLC2A4), and glycolytic enzymes (PFK, 6-phosphogluconate, and lactate dehydrogenase). This is proven by a study suggesting that the cumulus cells glycolytic level of glucose increases during oocyte maturation to meet the oocyte demand for energy, as evidenced by the higher level of glycolytic enzymes such as PFKP and PKM2 in mature oocytes compared to the immature oocytes group (P<0.05) (20, 40). Although the expression of PKM2 is higher in mature oocytes, this was not reflected in implanted embryo group (P<0.05). Similar findings were noted by Gebhardt who reported that the level of energy metabolites was not associated with embryo grading (12).

Besides glycolysis, glucose is also metabolized by several other pathways including the pentose phosphate pathway (PPP), hexosamine biosynthesis pathway (HBP), and polyol pathway. PPP is important for oocyte nuclear maturation. Products of PPP include NADPH, which is utilized for cytoplasmic integrity; reduction of glutathione, and phosphoribosyl pyrophosphate, which is an important control for nuclear maturation (78). Xie et al. (43) demonstrated that by applying RNA interference silencing of G6PD or GAPDH genes of cumulus cells (genes involved in PPP pathway), lower maturation rates were achieved in denuded oocytes cultured in the medium compared to those of control cumulus cells (43). Similar results were also obtained in another study on the pig oocyte model (44).

Synthesis of hyaluronic acid which mainly formed the backbone of ECM requires glucose metabolism. HAS2 metabolized glucose to hyaluronic acid *via* the hexosamine biosynthetic (HBP) pathway, with a key role enzyme glutamine-fructose-6-phosphate transaminase (GFPTs). Besides, HBP is also responsible for O-linked glycosylation for cell signaling. In contrast, Shen et al. reported the expression level of LDHA and GFPT were not associated with oocyte maturity, fertilization, embryo grade, and implantation.

And lastly, the polyol pathway provides an alternative energy production for the COCs through sorbitol and fructose substrate. This energy substrate is of limited use. Wongsrikeao et al. (79) showed that oocytes exposed to fructose-only IVM have less complete nuclear maturation compared to those cultured in the presence of glucose, but are helpful in the nuclear maturation process. Despite the importance of glucose metabolism in cytoplasmic and nuclear maturation, studies were showing the consequences of hyperglycaemic states, such as diabetes, obesity, and poor diet, leading to poor maternal health as well as resulting in poor oocyte viability (78).

In PCOS, glucose also plays a significant role in every stage of promoting oocyte maturation. Alteration in glucose metabolism in the peri-conceptional period has an impact on the pregnancy and the long-term health of the offspring. It is well established that PCOS women are mostly insulin resistant, thus resulting in a hyperglycaemic state. In addition, the presence of insulin-sensitive cumulus cells’ main glucose transporter, SLC2A4 worsened the condition. Hyperglycaemia results in reduced fecundity, increased miscarriage rates, and increased risk of congenital abnormalities. A study on mice with type 1 diabetes (hyperglycaemic state) showed that the oocytes have poor developmental competence due to poor folliculogenesis (80), defect oogenesis, and impaired oocyte maturation (81). In women with PCOS, it was demonstrated that PFKP and Ldha genes was significantly reduced due to excessive nerve growth factor (NGF) stimulation, hence resulting in decreased glycolysis and worsening the hyperglycemic state in PCOS women (41). NGF is a member of the neutrophin family and has been found to play important role in the reproductive system in addition to its’ normal function in the nervous system. Excessive NGF in PCOS women significantly inhibit oocyte meiotic maturation contributing to poor quality oocyte (41). There is also an alteration in follicular fluid composition in PCOS, especially with regards to the level of insulin, glucose, and lactate. The concentration of glucose in follicular fluid is comparable to plasma levels. Supplying too high or too low glucose levels, especially in the case of ART are detrimental to oocyte development (78). All these factors eventually lead to poor quality oocytes hence the low success rate of ART in PCOS women.

## Conclusions or Future Perspectives

This review has proved that the mechanism involved in determining oocyte quality and embryo development potential differ between PCOS and non-PCOS groups. For each, there were limiting factors that can cause controversial results and limit the endpoints of selecting a proper biomarker for the assessment of oocyte quality and selection. PCOS-related studies were much less and most are done on a small scale. However, worth mentioning that genes related to cumulus expansion such as HAS2, PTX3, GREM1, and VCAN were of great value to be used as potential oocyte markers in PCOS as it highly correlates with the regulation of oocyte signals and has important functions in cumulus expansion and metabolism (14, 23, 39). With proper methods, these genes can be utilized to complement current morphological assessment, thus improving the success rate of ART.

## Author Contributions

NS wrote the first draft of the manuscript, while MAA and MFA contributed to read, editing and revision of the final version. All authors contributed to the article and approved the submitted version.

## Conflict of Interest

The authors declare that the research was conducted in the absence of any commercial or financial relationships that could be construed as a potential conflict of interest.

## Publisher’s Note

All claims expressed in this article are solely those of the authors and do not necessarily represent those of their affiliated organizations, or those of the publisher, the editors and the reviewers. Any product that may be evaluated in this article, or claim that may be made by its manufacturer, is not guaranteed or endorsed by the publisher.
